# Hematopoiesis during Ontogenesis, Adult Life, and Aging

**DOI:** 10.3390/ijms22179231

**Published:** 2021-08-26

**Authors:** Alexander Belyavsky, Nataliya Petinati, Nina Drize

**Affiliations:** 1 Engelhardt Institute of Molecular Biology, Russian Academy of Sciences, 119991 Moscow, Russia; abelyavs@yahoo.com; 2 National Research Center for Hematology, 125167 Moscow, Russia; loel@mail.ru

**Keywords:** hematopoietic stem cell (HSC), mesenchymal stem cell (MSC), transcription factors, clonal hematopoiesis, bone marrow niche, aging

## Abstract

In the bone marrow of vertebrates, two types of stem cells coexist—hematopoietic stem cells (HSCs) and mesenchymal stem cells (MSCs). Hematopoiesis only occurs when these two stem cell types and their descendants interact. The descendants of HSCs supply the body with all the mature blood cells, while MSCs give rise to stromal cells that form a niche for HSCs and regulate the process of hematopoiesis. The studies of hematopoiesis were initially based on morphological observations, later extended by the use of physiological methods, and were subsequently augmented by massive application of sophisticated molecular techniques. The combination of these methods produced a wealth of new data on the organization and functional features of hematopoiesis in the ontogenesis of mammals and humans. This review summarizes the current views on hematopoiesis in mice and humans, discusses the development of blood elements and hematopoiesis in the embryo, and describes how the hematopoietic system works in the adult organism and how it changes during aging.

Hematopoiesis in vertebrates occurs in the bone marrow (BM) and is maintained throughout the life due to the coordinated functioning of two different stem cell types and their progeny: hematopoietic stem cells (HSCs) and mesenchymal stem cells (MSCs).

## 1. Development of Hematopoiesis in Embryo

### 1.1. Hematopoietic Cells during Embryogenesis

**Figure 1:**
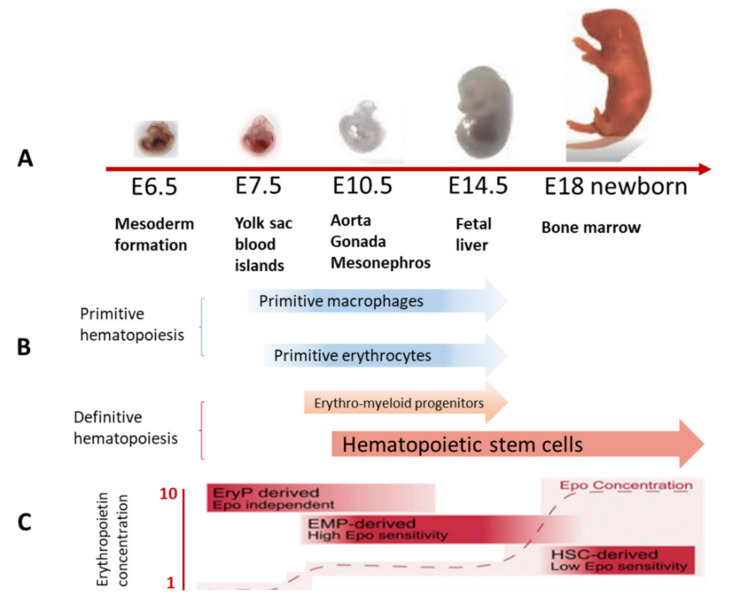
Hematopoiesis in the embryo. (**A**) Sequential change of sites of hematopoiesis in the embryo. (**B**) Timing of primitive and definitive hematopoiesis. (**C**) Megaloblastic erythroid precursors and fetal erythropoietin levels. Adapted from [3,8,9].

Embryonic hematopoiesis in vertebrates takes place at various locations (Figure 1A) and occurs in three successive stages (“waves”). The first two waves take place in the embryonic yolk sac outside the embryo proper, with the formation of transitional hematopoietic populations (megaloblastic hematopoiesis). The third wave arises inside the embryo in the region of the aorta–gonad–mesonephros (AGM), and gives rise to adult hematopoiesis (normoblastic hematopoiesis), resulting in the formation of HSCs and MSCs that provide the organism with continuous production of blood cells [1,2,3,4].

In the first wave of hematopoiesis, three types of blood cell are produced, namely primitive erythrocytes expressing embryonic globins, and megakaryocytes and macrophages [5,6,7]. Yolk sac erythroid progenitor cells but not HSC descendants maintain erythropoiesis throughout the mouse embryogenesis (Figure 1B) [8]. This is related to the fact that to efficiently produce erythrocytes, yolk sac-derived precursors require 10 times lower erythropoietin concentrations than their HSC-derived counterparts. In fetal liver environment with low erythropoietin content, yolk sac-derived erythrocyte precursors are likely to effectively displace HSC offspring that cannot generate megakaryocyte and erythrocyte precursors. In the absence of HSC activity, embryo viability prior to birth is maintained by hematopoietic cells derived from erythro-megakaryocytic progenitors (Figure 1C).

In the second and third embryonic waves, definitive multipotent cells are formed, namely erythromyeloid progenitors (EMPs) in the yolk sac blood islets, and hematopoietic stem/progenitor cells (HSPCs) in the dorsal aorta (DA), respectively. In these waves, a highly specialized endothelium type called hemogenic endothelium is formed, from which these hematopoietic precursors originate in a process known as endothelial-to-hematopoietic transition (EHT) [9,10,11]. Endothelial cells in the yolk sac and DA express either Tek or Ly6a genes. The formation of both EMPs in the yolk sac and HSCs in DA requires the participation of the transcription factor Runx1 and its partner, core-binding factor b (CBFb). Patterns of CBFb expression in EMPs and HSCs differ in time and space. Endothelial expression of CBFb in Tek-expressing cells is sufficient for EMP formation, but not adequate for HSC formation. On the other hand, expression of CBFb in cells expressing Ly6a is sufficient for the formation of HSCs, but not EMPs. Therefore, EMPs and HSCs arise from different populations of hemogenic endothelial cells, with Ly6a expression specifically marking HSC-generating hemogenic endothelium [12]. Moreover, expression of hepatic leukemia factor (Hlf) specifically marks developing HSCs in the embryo. Sequential upregulation in Hlf was observed in the fetal liver, but not in the AGM after E12.5 stage of embryonic development. *Hlf* is expressed in intra-aortic hematopoietic clusters and fetal liver (FL) HSCs, whereas EMPs and yolk sac hematopoietic clusters before embryonic day 9.5 do not express *Hlf*. Such specification further supports the notion that HSCs and EMPs are generated from distinct cohorts of hemogenic endothelium [13]. In an important study using single-cell transcriptomics, more than 20,000 cells immediately following gastrulation at E8 were analyzed. Twenty major cell types, which frequently contained subtypes, including three distinct signatures in early foregut cells, were described [14]. Within the endothelial population, cells that transition from hemogenic endothelial to erythro-myeloid progenitors specifically expressed Alox5 and its cofactor Alox5ap, leukotriene production controlling factors. Functional assays using mouse embryonic stem cells demonstrated that leukotrienes promote hematopoietic progenitor cell generation.

Following formation, EMPs and HSPCs migrate to the fetal liver and differentiate to form adult blood cells. Recent studies have identified the relationship between developing vascular and hematopoietic systems in animal models, including the molecular mechanisms that drive the endothelial-to-hematopoietic transition transcription program [15]. It has been shown that the aortic endothelium receives various morphogenetic signals along the dorsoventral axis, including regulators of BMP and WNT signaling pathways, which also includes additional intercellular interactions and regulation of the cell cycle. BMP signaling helps to establish the HSC niche in the DA region and is largely limited to the ventral side of the DA, where the subaortic mesenchyme produces BMP4. BMP signaling is further modulated by FGF, which is produced by dorsal somite tissue and represses BMP4 transcription while inducing BMP inhibitors such as noggin. As a result, HSC emergence is spatially restricted to the ventral portion of the aorta. Importantly, BMP signaling in the AGM is transient to enable the maturation of budding cells into functional HSCs. Accordingly, localized and transient BMP signaling is a prerequisite for correct spatiotemporal patterning of HSC emergence in the AGM [16]. Canonical WNT signaling is also required for the HSC specification in AGM, but is not necessary for the subsequent maintenance of emerging HSCs [15]. Via interaction with BMP and WNT signaling in the AGM, intercellular contact-dependent activation of NOTCH promotes hemogenic specification. In particular, DLL4/NOTCH1 signaling activates the arterial program, while JAG1/NOTCH1 signaling blocks it to trigger hemogenic specification. Although both DLL4 and JAG1 are expressed in the dorsal aorta, JAG1 has a higher affinity for the NOTCH1 receptor and functions at lower signaling levels, which helps to induce hemogenic identity [17].

Most of the pre-HSCs that form in the DA and yolk sac are immature, and these cells acquire the full transcriptional signature of the definitive HSC upon colonization of the embryonic liver only [18,19]. It is well established that epigenetic programming controls differentiation and is necessary for establishment of cell identity [20]. Among complex epigenetic regulations, chromatin availability is of primary importance for gene expression, since only the nucleosome-free DNA is available for transcription factors (TFs) [21]. TFs are critical effectors of regulatory gene cascades that determine cell fate. Fine tuning of chromatin availability results in the transition of HSPCs from immature to fetal state. In particular, Smarca5, a member of SWI/SNF family of epigenetic modifiers, interacts with nucleolin to promote chromatin remodeling and facilitate DNA binding of hematopoietic transcription factors in fetal HSPCs [22].

Among the many TFs functioning in the hematopoietic system, TAL1/SCL is the main hematopoietic transcriptional regulator. This protein is not only at the top of the hierarchy of TFs involved in hematopoietic specification, but is also essential for adult HSC function and for the terminal maturation of individual hematopoietic differentiation lineages. SCL functions in the early stages of blood development. Embryos with SCL knockout die on the 9.5th day of development due to the lack of primitive erythropoiesis and myelopoiesis in the yolk sac [23]. All definitive adult hematopoietic clones (wave 3) were absent in SCL^−/−^ chimeras [24]. SCL associates with a large variety of protein partners to establish activating and repressive transcriptional complexes in a lineage- and stage-specific manner. During development, SCL is essential for (1) specification of three hematopoietic waves, (2) maturation of select blood lineages, and (3) remodeling of the vascular network [25]. Expression of another TF, Runx1, plays an important role in embryogenesis, in particular during the time when the earliest blood cells are formed, with its expression slightly preceding the appearance of blood cells. In the yolk sac, Runx1 is expressed in the mesoderm of putative blood islets beginning at stage E7.5. Differentiation of blood islet mesoderm into primitive erythrocytes stops shortly after the appearance of these cells [26]. Runx1 is expressed in all emerging HSPCs in hematopoietic clusters of the vascular system [27]. Its expression is also found in subaortic mesenchymal cells starting at the E10 stage. These cells coexpress smooth muscle actin, suggesting that they develop into smooth muscle cells [28].

It should finally be noted that fetal and adult HSPCs demonstrate significant differences in several key functional aspects such as cell cycle kinetics [29,30,31], response to inflammatory stress, and the production of various subpopulations of blood and immune cells [32].

### 1.2. Mesenchymal Cells during Embryogenesis

Starting with early ontogenesis, hematopoietic precursors develop in an ensemble with mesenchymal cells, as evidenced by many findings. Mesenchymal precursors, which can differentiate into cells of osteogenic, adipogenic, and chondrogenic lineages, are localized in most areas harboring hematopoietic cells. They first appear in the AGM area during the appearance of the HSC. However, at this stage the presence of mesenchymal precursors does not depend on HSC activity. Their number increases during development to the plateau level found in adult bone marrow. In addition, mesenchymal precursors are found in the embryonic bloodstream. Taken together, these data show colocalization of mesenchymal progenitor/stem cells in major hematopoietic sites, suggesting that, as development progresses, there is an increase in mesenchymal progenitors in these important sites of hematopoiesis [2]. Moreover, in adult animals, in which hematopoiesis occurs only in the bone marrow, foci of extramedular hematopoiesis arising in pathological conditions are localized only in places where embryonic hematopoiesis took place. This indicates that mesenchymal stromal cells organize the microenvironment for hematopoietic progenitors. Most likely, the mesenchymal stem cells established during embryogenesis may under certain conditions “recall” their ability to support hematopoiesis.

Immediately after birth, hematopoiesis occurs in the bone marrow. HSCs are located in niches mainly formed by MSC and their descendants, and their function is associated with extracellular matrix molecules, hematopoietic cytokines, and chemokines.

It should finally be noted that cell migration and repopulation are heavily involved in the functioning of the hematopoietic system. In the early fetal development, movement of hematopoietic cells in the embryo occurs due to morphogenetic movements, whereas after the closure of the blood circulation loop, the intravascular migration is involved in changing the sites of hematopoiesis. In an adult organism, there is a constant migration and repopulation of hematopoietic cells.

## 2. Maintenance of Hematopoiesis in the Adults

### 2.1. Hematopoietic Cells in Adult Life

The enormous quantity of blood cells produced every day in humans, about one trillion [33], requires a large number of HSCs to function simultaneously. Based on the analysis of numerous data, it can be assumed that less than 1.3 million HSCs support the formation of mature peripheral blood cells in a person’s life [34]. Estimates of the number of functioning HSCs in healthy people are based on indirect evidence (for example, the detection of somatic mutations in the offspring of HSCs in one person) [35] and range from as few as 385 active HSCs [36] to 50,000 to 600,000 HSCs in steady state adult hematopoiesis [34,37]. Since the progeny of one HSC is a clone [38], stable hematopoiesis is normally polyclonal, i.e., many HSCs are simultaneously involved in the production of peripheral blood cells [39,40,41]. The combination of genetic barcoding and next-generation sequencing (NGS) methods made it possible to determine the most complete clonal composition of the hematopoietic cell population and its dynamics in the hematopoietic system of mice, primates, and humans after transplantation of marked bone marrow cells into an irradiated or appropriately conditioned organism [42,43,44,45,46]. These studies confirmed the earlier proposed clonal succession model stating that many successive hematopoietic cell clones support hematopoiesis. The use of such a sensitive method of labeling the HSC population isolated by cell sorting demonstrated that the majority of transplanted clones continuously contribute to hematopoiesis for a long time, although the clonal composition of granulocytes, T cells, and B cells differs significantly. The contribution of individual clones to hematopoiesis is constantly changing [47]. Importantly, clone sets in peripheral blood and bone marrow may differ in part. The contribution of a given clone changes over time in mice, although many clones can be observed for more than 12 weeks. The data obtained demonstrate an interesting phenomenon: lymphocytes and granulocytes often bear nonidentical barcodes, which indicates that the transplanted HSC populations, despite their apparent phenotype homogeneity, are nevertheless heterogeneous and consist of cells biased to lymphoid or myeloid differentiation [48,49]. This agrees with the results of other studies in which an entirely different research method was used. In humans, a consecutive exit of HSCs from the dormant state to the phase of active division and differentiation was found for five years without returning to the resting phase [45,50]. Hundreds of HSC clones have been found in humans and primates, the offspring of which is present in different hematopoietic differentiation lineages. Many clones and clonal instability were observed during the first year after transplantation, followed by system stabilization [44].

Nearly all the current knowledge on the polyclonality of hematopoiesis has been based so far on the transplantation of labeled cells into irradiated organisms. Another approach involving individual labeling of cells in vivo that does not require subsequent transplantation is based on the use of a transposon system established in transgenic mice [51,52]. In this method, due to activation of the “Sleeping Beauty” transposase by tetracycline, the integrated transposon is moved to a random place in the genome, and this place is unique for each cell and is inherited by cell descendants. Cells derived from a common progenitor are characterized by the common location of the transposon. The analysis of transposon localizations demonstrated the results that were strikingly different from the previous studies. Namely, at each time point in the study, completely different clones turned out to support hematopoiesis. According to the authors’ estimates, thousands of clones function simultaneously in the mouse. Thus, these data indicate that long-term hematopoiesis is supported by the successive change of a large number of clones.

Although the observed clonal dynamics is consistent with the clonal succession model, the putative clonal diversity is much higher than the one previously demonstrated by transplantation experiments. In the same study, when the bone marrow of mice with relocated transposons was transplanted into irradiated recipients, comparison of hematopoietic clonal composition in donors and recipients failed to reveal identical clones. Further, when authors compared the clonal composition of HSCs, intermediate progenitors, and mature cells in each mouse, less than 5% of HSC clones were present in mature cell populations, while the main participants in the production of mature cells were intermediate multipotent (MPP) and myeloid (MyP) precursors. Based on these two experiments, the authors concluded that cells that provide stable hematopoiesis under normal conditions are incapable of transplantation and therefore are not found in the total HSC pool. These results indicate that the maintenance of normal hematopoiesis under steady-state conditions is primary driven by a large number of progenitor cells, including MPPs and MyPs. However, these results do not exclude the possibility of HSC involvement in stable hematopoiesis.

**Figure 2:**
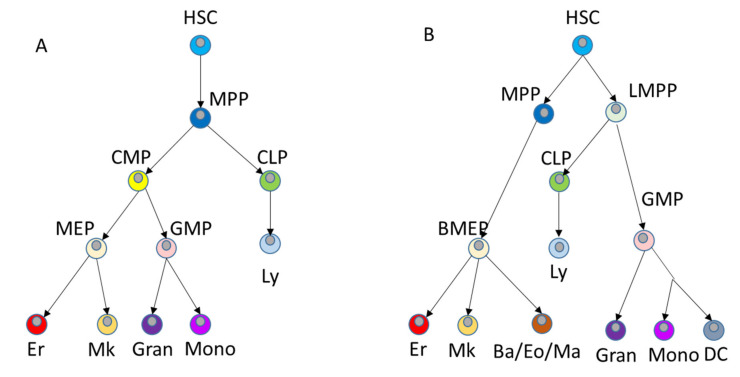
Hierarchical models of hematopoiesis: (**A**) 2006 model, adapted from [60]; (**B**) modern model of continuous hematopoiesis, adapted from [59]. MPP—intermediate multipotent progenitors, CMP—common myeloid progenitors, CLP—common lymphoid progenitors, MEP—megakaryocyte/erytroid progenitors, GMP—granulocyte-macrophage progenitors, Er—erythrocytes, Mk—megakaryocytes, Gran—granulocytes, Mono—monocytes—macrophages, LMPP—lympho-myeloid polypotent progenitors, Ba—basophils, Eo—eosinophils, Ma—macrophages, Ly—lumphocytes, DC—dendritic cells.

In accordance with the fact that the transplanted HSCs are located in the bone marrow and are normally resting, HSCs are minimally involved in the formation of differentiated blood cells during stable hematopoiesis. The use of molecular barcoding systems has established that during steady-state hematopoiesis, megakaryocytes can derive from HSCs, bypassing in this case the stage of common myeloid precursors (CMP) [53,54]. A similar conclusion regarding the existence of megakaryocyte differentiation-specific HSCs has been reached in a work by Carrelha et al. using an entirely different approach based on single cell transplantation [41]. A study by Pei et al. using a distinct in situ barcoding system revealed that differentiation inactive, multilineage, and lineage-restricted HSC clones reside in distinct regions of the hematopoietic transcriptional landscape [55]. Pei et al. did not report existence of megakaryocyte-specialized HSCs, presumably because megakaryocyte lineage was not sampled in their work. Studies of the differentiation of individual HSCs revealed that under homeostatic conditions, all HSC clones uniformly differentiate into all hematopoietic cell lines. In contrast, when the hematopoietic system is disturbed by irradiation or antagonist antibodies against c-Kit, only a small fraction of donor HSC clones differentiate [56]. Based on the new data, the sustainable hierarchy model underwent a revision [57,58]. Analysis of single cell RNA signatures of about 200,000 BM cells made it possible to identify 26 distinct cell populations in healthy people and create a comprehensive atlas of these populations that can be used as hematopoietic reference [59]. As a result, the authors built a continuous hierarchical model of hematopoietic cells maintaining normal hematopoiesis (Figure 2B).

During the normal balanced hematopoiesis, most HSCs are dormant. In addition to maintaining homeostasis and hematopoietic tissue repair, these cells are also capable of self-renewal to maintain a resident stem cell population [61]. Dormant HSCs have exited the cell cycle and entered the G0, but can re-enter it to proliferate and differentiate in response to various stimuli and stressors [62]. Quiescent HSCs, which are located in unique and specialized niches, can have different resting profiles, but in general are characterized by tight regulation of all cellular processes. Some of the key factors and processes that have been studied in the context of the dynamics and maintenance of cell dormancy are presented in Table 1 [63].

In addition, quiescent HSCs have a low content of reactive oxygen species (ROS) [64,65], which is necessary to maintain their repopulation potential. With stable hematopoiesis, ROS levels in HSC fluctuate in accordance with circadian rhythms (light/dark signals) [66]. HSCs are dormant for long periods and do not divide very often. It is estimated that HSCs divide only five times during a mouse’s life [67,68].

### 2.2. Mesenchymal Cells in Adult Life

In recent years, it has become increasingly clear that the environment in which HSCs are located has a profound impact on their biology and behavior. More specifically, HSCs in bone marrow are associated with the stromal microenvironment termed “niches”, which support and regulate stem cell function through cellular interactions and secreted factors [69,70]. Niche formation involves a number of nonhematopoietic stromal cells (mesenchymal stem cells (MSC), osteoblasts, adipocytes, CXCL12-producing cells, fibroblasts) [71], endothelial cells [72], osteoclasts, macrophages [69], and nonmyelinating Schwann cells [73,74].

One of the main components of the niche are multipotent mesenchymal stem/stromal cells (MSCs), nonhematopoietic cells derived from the mesoderm with the potential to differentiate into bone, adipose, and cartilaginous tissues in vitro [75]. Although MSCs are found in most tissues, their diversity and relationships are not well understood. For example, several MSC subtypes that regulate HSC are described in specialized bone marrow niches. Most MSCs reside in the perivascular space and are associated with arterioles or sinusoidal blood vessels and produce key HSC niche factors such as the chemokine Cxcl12 and stem cell factor (SCF, also named KitL) [70]. They are variously identified as leptin receptor-positive cells [76], Nestin-positive cells [77], and NG2 pericytes [78]. Ablation studies have shown that MSC populations expressing Nestin, Cxcl12, SCF, and leptin receptor are required for the maintenance and differentiation of blood cells [76,79,80]). It was further shown that hematopoietic progenitor cells are predominantly localized in the immediate vicinity of MSCs that secrete key factors associated with the HSC maintenance, and next to small arterioles or sinusoidal endothelium [77,81,82].

The main characteristics of MSCs and their descendants (multipotent mesenchymal stromal cells—MMSCs) have been described in in vitro studies. These cells are currently receiving substantial attention due to their increasing use in regenerative medicine, not only because of their ability to differentiate into certain types of cells, but also, more importantly, due to their paracrine effects [83]. Their cross-interaction with the immune system makes MMSCs ideal candidates for use in the treatment of autoimmune diseases or graft versus host disease [84], or as carriers in anticancer therapy [85]. MMSCs is a heterogeneous population, and is represented by cells that differ in proliferative potential [86,87,88,89,90]. When MMSCs are passaged in culture, the clonal composition of population changes significantly due to the exhaustion of the proliferative potential of the majority of cells. Large clones were found only at early passages. The polyclonal population of MMSCs contains only a small number of cells with a high proliferative potential, potentially including stem cells [91]. Differentiation capacity of MMSC clones also varies [87].

**Figure 3:**
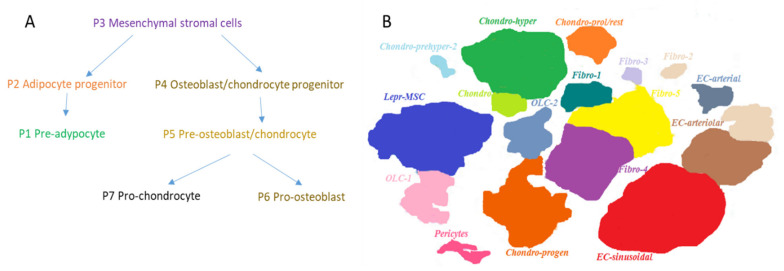
Population structure of the mouse BM stroma compartment identified by single-cell transcriptome analysis: (**A**) distinct BM niche populations and their differentiation paths revealed by SPRING plot of single-cell transcriptomes, adapted from [90]; (**B**) cellular taxonomy of BM stroma and seventeen BM stroma cell clusters identified by t-Distributed stochastic neighbor embedding (t-SNE), adapted from [92]. Colors indicate graph-based cluster assignments.

MSC offspring also takes an active part in the regulation and maintenance of hematopoiesis. To analyze unique cellular subpopulations in the bone marrow, single-cell RNA sequencing of nonhematopoietic bone marrow cells during homeostasis was performed [91]. By combining computational prediction of the cell state hierarchy with the known expression of key TFs, the differentiation pathways of osteocyte, chondrocyte, and adipocyte lineages were compared. This analysis allowed identification of gene signatures of specific subpopulations together with prediction and confirmation of TFs that mediate differentiation of stromal cells. This work confirmed the existence of a simple branching differentiation hierarchy in MSC compartment. Several TFs control the fate of specific stromal bone marrow lineages. These results were confirmed using transgenic reporter mice strains and by studies of cell differentiation potential in culture. Seven subpopulations of stromal cells were identified. To identify the genes enriched in each subpopulation, a gene enrichment analysis was used to characterize the most significant gene expression signatures from each cell state. Certain subpopulations expressed genes associated with cell adhesion, cytokine production, HSC support, adipogenesis, and osteogenesis. Individual genes were also expressed in the expected manner, in distinct subpopulations expressing stromal (e.g., Cxcl12, KitL) or bone-associated (e.g., Bglap, Col1a1) genes at high levels. Visualization of single-cell transcriptomes using the SPRING method [53] revealed a continuum of cell states that group into two major branches reflecting a previously identified differentiation process (Figure 3A). Both branches originate from the MSC cluster (P3) located at the top of the diagram, and diverge into the branch containing adipocyte precursor (P2) and pre-adapocyte (P1) clusters, and the branch harboring osteoblast and chondroblast precursors (P4) that are followed by pre-osteoblast and pre-chondrocyte cluster (P5), which itself diverges into pro-osteoblasts (P6) and chondrocytes clusters (P7).

In another study, bone marrow stromal cells were analyzed using single-cell RNA sequencing in combination with a different method of data presentation, namely t-distributed stochastic neighbor embedding (t-SNE) [92]. Based on gene signatures, the authors identified 17 subpopulations of stromal cells (Figure 3B).

In the studies noted above, all populations of stromal cells were characterized in great detail, and the main TFs that determine the identified clusters of cells forming the stromal microenvironment of hematopoietic progenitor cells coincided. The expression of genes highly indicative of niche cells has been also confirmed in both works. In the study [91], Prrx1, Cxcl12, Lepr, Nt5E, and Runx2 genes were found to be highly expressed in MSCs, whereas in [92], this list contained Cxcl12, Lepr, Kitl, and Angpt1. Expression of these genes reported previously as involved in HSC regulation confirms the conclusions of earlier studies [70,93,94,95]. Comprehensive single-cell analysis thus provides the framework for a better understanding of the transcriptional networks regulating differentiation of bone marrow cells, functional features of HSCs, MSCs, and organization of osteoblastic and vascular niches [96,97,98,99].

MSC differentiation is of primary importance for maintaining normal hematopoiesis and is controlled by a complex signaling network. Runx2 and Osterix are key transcription factors for osteogenic differentiation, while PPARγ and CEBPα for adipogenic differentiation. Stress-related factors directly affect MSC differentiation. Aging, radiation, and chemotherapy can trigger a number of events that regulate the key transcription factors Runx2 and PPARγ, leading to a shift in the adipo-osteogenic balance.

The existence of connection between hematopoiesis and adipocyte differentiation in the bone marrow was observed long ago both in experimental and clinical settings [99]. In the areas of active hematopoiesis in the red bone marrow, the amount of lipid droplets in adipocytes is significantly reduced in comparison to yellow bone marrow. In contrast, under severe myelosuppressive conditions such as aplastic anemia or irradiation that damage hematopoietic tissues, the lipid content in adipocytes increases, leading to the shift towards white marrow that is rich in adipose tissue and does not support hematopoiesis [100]. These findings led to a belief that osteogenesis promotes hematopoiesis, while adipocytes represent negative regulators of hematopoiesis [101]. Subsequently, this notion has been confirmed by demonstrations both in vivo and in vitro that bone marrow adipocytes act as negative regulators of hematopoiesis and inhibit the growth of hematopoietic cells [102]. The balance between osteogenesis and adipogenesis thus affects hematopoiesis and depends on shifts in MSC differentiation [103]. Additionally, an increase in the number of adipocytes in the bone marrow contributes to enhanced formation of osteoclasts that carry out bone resorption [104], which in turn results in a decrease in a number of HSC niches.

## 3. Aging in the Hematopoietic System

### 3.1. Hematopoietic Cells during Aging

Aging in vertebrates is accompanied by a marked decline in the function of the hematopoietic and immune systems, which leads to an increased risk of infection, poor response to vaccinations, anemia, increased risk of bone marrow failure, and the development of hematological tumors—leukemia and lymphomas [105,106]. To fully understand how aging affects hematopoiesis, it is necessary to know how aging alters the biology of HSC and MSC, and how the hematopoietic system compensates its diminished function. An additional aspect in the HSC aging is the fact that HSCs exist in the bone marrow microenvironment, which includes many different types of hematopoietic and nonhematopoietic cells, as well as secreted factors, all of which also accumulate aging-related changes [107]. Aging in the hematopoietic system is thus caused by changes in both the internal properties of HSCs and external signals they receive from the aging organism (Table 2).

Although HSCs provide balanced production of all blood cells throughout the majority of the lifetime, they gradually lose their ability for self-renewal and regeneration with age. One of the most important manifestations of aging is stem cell functional depletion, which refers to the gradual decline in ability of adult tissue-specific stem cells to maintain cellular homeostasis of the tissue in which they reside. A conceptually important study has recently suggested that the most primitive, rarely dividing HSCs can only undergo four to five symmetric divisions during the entire lifespan of a mouse [68]. This study implied that HSCs somehow keep track of their divisions and that the age-related HSC functional changes including the loss of capacity for self-renewal and long-term repopulation occur primarily after the HSC divides five times. It should be noted, however, that the recent work by Morcos et al. [108] questioned the validity of conclusions by Bernitz et al., demonstrating considerable accumulation of background fluorescence in aged HSCs due to leaky fluorescent H2B histone expression, which was misinterpreted as stable retention of label. A part of the supposed timing mechanism may be represented by telomeres that progressively shorten with age in human HSCs isolated from fetal liver, umbilical cord blood, or adult bone marrow in parallel with greatly reduced proliferative potential [109]. Taken together, these data suggest that the task of proliferation and cell replenishment is performed primarily by committed progenitors, and that during stable hematopoiesis the most primitive stem cells are largely inactive. It seems likely that with each cell division, the proliferative potential of the HSCs decreases, but this is accompanied by the expansion of HSC pool via symmetric divisions as a compensatory mechanism for the functional decline of individual stem cells [110]. An age-related increase in the frequency of putative stem cells, coinciding with functional impairment and a decrease in lymphoid potential, has been reported in mice and humans [111,112,113]. Age-related defects in lymphoid potential [114] and lymphopoiesis [115] are thought to underlie the dominance of myeloid cells in adult leukemia.

The homing efficiency of young HSPCs is two- to three-fold lower in old mice as compared to young ones, and in the young mice, the aged HSPCs demonstrate a similar reduction in homing efficiency [116]. Noteworthy, the latter finding has been disputed by Verovskaya et al. [47] who did not observed reduction in HSPCs homing upon aging; the reasons for this discrepancy remain unclear. In contrast to homing, the G-CSF-induced mobilization of HSCs into the bloodstream is enhanced in aged mice as compared to young ones [117]. This correlates with decreased adhesion of the immature cell population (nonpurified HSC population) to stromal cells and with increased activation of Cdc42, a small Rho GTPase.

All studies that have evaluated old and young HSCs so far have shown a marked shift of HSCs toward myeloid versus lymphoid differentiation in aged mice [118]. Multiple single-cell RNA sequencing studies have been performed to elucidate how the heterogeneity and aging of HSCs are related [41,119,120]. Combination of functional and transcriptome analyses at the level of individual cells revealed that with age, a bias for increased platelet production by HSCs is observed, accompanied by expansion of a specific platelet-primed HSC subset. Importantly, suppression of platelet programming in mice induced by ablation of the transcription factor FOG-1 was accompanied by expansion of lymphoid compartment. Conversely, it is plausible that the age-related shift towards platelet differentiation might contribute to the concomitant decline in lymphopoiesis [120].

The numerous differences between young and old HSCs are summarized in Table 3.

As vertebrates age, their tissues accumulate an increasing number of somatic mutations. Rare mutations that increase cell fitness and provide an advantage to clonal growth are called driver mutations. Most of the other mutations are neutral, providing no fitness advantage to cells bearing them, and are thus called passenger mutations. However, these mutations can also take part in clonal evolution, in particular through a phenomenon called genetic drift, in which allele frequencies of the mutation change over time due to mere chance [122]. In addition, when passenger mutations occur in the same cells as the driver’s mutations, the passenger mutations increase their allele frequency along with the driver’s mutations [123].

A prominent role of somatic mutagenesis in aging processes applies to hematopoiesis as well. Aging in the mammalian hematopoietic system is accompanied by drastic changes of its clonal structure: polyclonal hematopoiesis of young age gradually gives way to the oligoclonal one, and may eventually evolve into clonal hematopoiesis (CH). The latter is characterized by predominance in the hematopoietic system of one or several clones, in most cases containing somatic driver mutations in a limited number of genes. A substantial proportion of circulating blood cells at the advanced age may thus derive from a single mutated stem cell [124]. Importantly, CH is a biological condition that does not normally result in overt pathologies. Therefore, it fundamentally differs from leukemia and lymphoma, which are characterized by expansion of clonal cells that are unable to differentiate and thus perturb and inhibit normal hematopoiesis [125]. In general, the accumulation of DNA damage decreases the function and maintenance of HSCs as they age. However, driver mutations in HSCs leading to CH may also give rise to overproduction of mutated immune effector cells, such as monocytes, granulocytes, and lymphocytes. These effector cells can potentially influence many disease states, especially those with a chronic inflammatory component [126], resulting in elevated disease risk. In polycythemia vera and essential thrombocytemia, blood diseases belonging to a rare type of CH with pathological manifestations [127], overproduction of erythrocytes and thrombocytes results in a highly increased incidence of thrombotic events [128].

A number of recent large-scale studies of hematopoiesis in elderly fully support the notion of reduced clonality and clonal hematopoiesis at advanced age. Full-exome deep sequencing of peripheral blood cells in 12,380 persons [129] and 17,182 persons [130] identified somatic mutations on the basis of unusual allelic fractions. The dominance of a single clone with somatic mutations was detected in 10% of people over 65 in the first study and in 9.5% of people over 70 in the second one. These results clearly demonstrate the replacement of polyclonal hematopoiesis with oligoclonal one with age, other authors demonstrated that people over 90 years old have oligoclonal hematopoiesis [109,129,130]. Deep sequencing of the entire genome revealed approximately 450 somatic mutations in noncoding AT-rich regions in the genome of normal hematopoietic cells in a 115-year-old woman [109]. The frequency distribution of allele variants of these mutations suggested that the majority of peripheral leukocytes were descendants of two related HSC clones. It is noteworthy that in the same study, telomeres in leukocytes were significantly shorter than in cells of other tissues. It seems plausible that the number of HSCs created during early development is limited and may not be sufficient to maintain hematopoiesis in centenarians. In this case, both the effects of somatic mutations and the finite lifespan of HSCs may be the major drivers of clonal evolution at extreme ages.

Under conditions of chronic stress, including bone marrow transplantation, chronic inflammation, genotoxic stress (chemotherapy), and aging, HSCs are continually induced to proliferate, which accelerates their exhaustion. Recent evidence demonstrates that inactivation of the inhibitor of DNA-binding gene 1 (Id1) can protect HSCs from depletion during chronic proliferative stress by promoting their quiescence [131]. During human aging and under proliferative or inflammatory stress, HSCs show increased DNA damage and DNA damage response during normal self-renewal and differentiation [132,133,134]. Older adults have increased DNA damage and double-strand breaks in CD34^+^ HSCs compared to younger persons [135,136]. DNA damage in HSCs is associated with a deficit in stress response, decreased proliferative potential, depletion of HSCs, and limited potential for hematopoiesis restoration in BM recipients from an elderly donor [137,138,139]. In general, the accumulation of DNA damage decreases the function and maintenance of HSCs as they age [131].

### 3.2. Mesenchymal Cells during Aging

Bone marrow aging is a complex process that is involved in the progression of many diseases, including hematological malignancies. The existing experimental data in this field emphasize the importance of cross-talk between HSCs and MSCs. Aging of MSCs not only affects their behavior but also has a direct impact on hematopoiesis [107]. A clear link between aging, remodeling of the bone marrow microenvironment, and impaired hematopoiesis has been previously demonstrated [140]. MSCs produce a large number of extracellular vesicles (EVs), with small vesicles playing an important role in cell-to-cell communication. EVs transport various elements such as proteins, lipids, and microRNAs to target cells and are involved in many biological functions of MSCs [141,142]. Aging of MSCs alters production of EVs and has a direct impact on their function and ability to differentiate [143].

MSCs can only undergo a limited number of cell divisions before entering senescence. Many factors have been described that cause MSC aging, such as oxidative stress [144], shortening of telomeres that occurs during in vitro expansion, and irreparable damage to DNA [107]. The accumulation of senescent cells in aging tissues has also been reported, in particular in a recent study evaluating the expression of p16 and p21, important senescence markers, in donors of various ages [145]. Elevated p21 levels have also been observed in bone marrow MSCs in the elderly, suggesting that senescent MSCs accumulate with physiological aging. Several pathways participating in cell cycle arrest in senescent MSC have been identified, namely the well-established p53/p21 and p16/pRB pathways, the AKT/mTOR pathway, the AK/STAT pathway, and pathways involving mitogen-activated protein kinase p38MAP and fibroblast growth factor FGF21 [107,146]. Aging cells undergo epigenetic modifications, which is accompanied by many cellular alterations at the morphological and functional levels. In particular, cells enlarge and their resistance to apoptosis increases while the autophagy declines. With aging, MSCs exhibit increased secretion of growth factors, pro-angiogenic factors, extracellular matrix remodeling factors, and, in particular, pro-inflammatory cytokines such as IL-1β, IL-6 and IL-8 [147,148,149]. Epigenetic modifications are key components of bone marrow niche homeostasis and may contribute to age- and disease- related MSC changes. Modifications of DNA methylation patterns and appearance of hypermethylated and hypomethylated CpG sites at several genomic loci have been observed during replicative senescence in MSCs [150]. Sirt1, a NAD-dependent histone deacetylase, acts to protect MSCs from depletion and maintain their differentiation potential during aging [151]; however, its expression and activity decreases with age. Epigenetic modifications of MSCs that occur during aging may contribute to abnormalities in hematopoiesis. For instance, inhibition of DNMT1 and DNMT3b induces cellular senescence [152] in human cord blood MSCs (UC-MSCs), the loss of TET1 and TET2 impairs self-renewal and differentiation of MSCs [153], whereas inactivation of ASXL1 disrupts the fate of MSCs and their ability to support hematopoiesis [154].

A direct connection of aging hematopoiesis with age-related changes in bone and cartilaginous tissues has been established [155]. Moreover, it has become clear that the aging of bones has an earlier onset than the age-related decline in the hematopoietic and immune systems, confirming the idea that changes in bones and bone marrow may be the basis on which functional reduction of HSCs occurs [104]. In human MSCs, significant age-related differential expression of HEXA, HEXB, CTSK, SULF1, ADAMTS5, SPP1, COL8A2, GPNMB, TNFAIP6, and RPL29 genes was found; importantly, these genes have been earlier implicated in bone loss and the pathology of osteoporosis and osteoarthritis with aging [156]. A number of substantial modifications in BM niches during aging have also been reported (Table 4).

## 4. Mechanisms of HSC Aging

### 4.1. Oxidative Stress and DNA Damage

Quiescent HSCs residing in hypoxic BM niches are protected from oxidative stress and, in addition, keep their mitochondria relatively inactive by using glycolysis for energy supply [157]. Low mitochondrial activity is important for maintaining HSC stemness [158]. However, leaving quiescent state and starting cell division is accompanied by switch to oxidative phosphorylation, increased mitochondrial activity and ROS production, which is necessary for HSC differentiation [159]. ROS are well known for inflicting damage in DNA and inducing senescence, and thus when HSCs enter cell cycle to perform their main function they are inevitably subjected to the increased risk of damaging their DNA, as directly demonstrated by Walter et al. [134]. In the aged HSCs, oxidative stress is substantially elevated compared young ones, and DNA damage is several-fold higher [160,161]. In addition, ROS levels are increasing in BM niches during aging [162]. Thus, combination of HSC-intrinsic and extrinsic ROS elevation is likely to contribute both to expansion of HSCs and to their increasing malfunction during aging.

Essential role of DNA damage in HSC aging has been suggested by premature HSC aging in mice with defects in DNA repair mechanisms [160,163]. Since quiescent HSCs were shown to use error-prone nonhomologous end joining repair mechanism [164], increasing niche ROS levels may represent an additional factor inducing DNA damage and disfunction in aged HSCs.

### 4.2. Mitochondrial Malfunction

Maintaining optimal mitochondrial function and health is of critical importance for HSC biology. Excessive mitochondrial activity is accompanied by increased proliferation and loss of regenerative potential by HSCs as well as premature aging, whereas induction of quality control programs such as mitochondrial unfolded protein response and mitophagy is instrumental in counteracting detrimental effects connected with mitochondria during aging [165,166,167]. Signaling axes involved in nuclear-mitochondrial communication and control of mitochondria state, namely NAD+-Sirt1-HIF-1α, Foxo-Sirt3-antioxidant enzymes, and Sirt7-NRF1, have been implicated in maintenance of HSC function (reviewed in [168]). Importance of NAD+ levels in controlling activity of mitochondria and their clearance has been highlighted by recent demonstration that supplementation of NAD+-precursor nicotinamide riboside significantly enhances hematopoiesis in mice [169].

### 4.3. Defects in Proteostasis

Another important mechanism helping to maintain optimal fitness in cells and tissue is protein homeostasis, or proteostasis. Several specialized types of proteostasis contribute to its implementation, in particular the proteasome system, the unfolded protein control system and autophagy, as well tight control of protein synthesis and modification [170]. Failure to maintain optimal proteostasis leads to accumulation of damaged or misfolded proteins and significantly contributes to aging phenomena. In the hematopoietic system, the proteasome system controls levels of a number of proteins critically involved in HSC function [171], whereas unfolded protein response contributes to hematopoietic regeneration [172]. Autophagy is very important for maintenance of HSC function and health, especially in aging cells. Thus, knockout of essential autophagy gene Atg7 in the hematopoietic system resulted in increased ROS levels, excessive proliferation, and loss of HSC function [173]. Substantial levels of autophagy are retained by aging HSCs, whereas loss of autophagy results in accumulation of mitochondria in activated metabolic state, induces myeloid shift and impairs self-renewal activity in HSCs [174,175]. The critical importance of protein homeostasis in aging is coming to the forefront in the last few years and, despite the results mentioned, there is still an apparent paucity of HSC-relevant data in this field, necessitating further investigation of the role of proteostasis in HSC aging.

### 4.4. Polarity Loss

One of the important mechanisms contributing to HSC aging is asymmetry of cell division. In yeast, damaged and aggregated proteins are distributed during the asymmetric division to mother cells, which are thus destined to undergo gradual aging, whereas daughter cells stay youthful [176,177]. This mechanism seems to be retained, at least to some extent, by multicellular organisms. For young HSCs with little or no replication history, division is reportedly asymmetric, whereby one of daughter cells receives a set of healthier, functional organelles and conserves a higher regenerative potential than the other, less “lucky” cell, which gets organelles of reduced quality and becomes more differentiated with diminished HSC functionality [178,179,180]. This process is associated with polarity in HSCs themselves that is lost during aging, with corresponding replacement of asymmetric division by a symmetric one [181]. The HSC polarity is in turn controlled by the small GTPase CDC42. Higher levels of CDC42 activity are observed in aging HSCs and causally linked to their polarity loss and functional decline [182].

### 4.5. Deregulation of Epigenetic Mechanisms

Cell differentiation does not normally involve changes in DNA sequence and thus is inevitably regulated by epigenetic mechanisms. This is turn highlights the importance of epigenetic machinery in maintenance of the undifferentiated HSC state and implicates epigenetic modifications as responsible for long-term functional changes during HSC aging. Transcriptome analyses revealed upregulation of genes associated with specification of myeloid fate and function, and stress response, inflammation, and protein aggregation in aged HSCs, with concomitant downregulation of genes involved in lymphoid specification and function, preservation of genomic integrity, and chromatin remodeling [183,184]. Both HSC functional decline and changes in DNA methylation patterns during aging, in particular hypermethylation of genes regulated by Polycomb Repressive Complex2 (PRC2), were found to depend on the proliferative history of HSCs [185]. DNA methylation is necessary for lymphoid lineage choice during HSC differentiation [186]. Importance of epigenetic modifications in HSC function is evidenced by knockout studies of genes participating on DNA methylation or hydroxymethylation. Thus, inactivation of de novo DNA methylase Dnmt3a and, to lesser extent, Dnmt3b, enhances HSC self-renewal and blocks their differentiation [187,188]. Depletion of Tet2 enzyme performing hydroxymethylation of DNA promotes self-renewal and expansion of HSCs, as well as myeloid bias and myeloid malignancies [189,190]. Importantly, DNMT3A and TET2 are two genes most frequently mutated in hematopoietic cells of individuals with clonal hematopoiesis [191]. Moreover, inactivation of a number of genes participating in PRC1 or PRC2 complexes, such as Bmi-1, Ezh1, and Suz12, and various histone lysine acetyltransferases results in HSC exhaustion (reviewed in [168]). Aging also induces rearrangements in chromatin structure that are manifested in the loss of polarity of certain histone H4 modifications and changes in chromosome 11 architecture that are connected to the levels of Cdc42 activity and may be reversed by lowering the levels of the latter [192]. Despite the compelling evidence of the importance of epigenetic factors both in HSC function and aging-related decline, the major driving forces behind the age-associated epigenetic alterations remain to be established.

## 5. Perspectives of Rejuvenation of the Hematopoietic System

One of the important questions arising in connection to aging is whether it is possible to delay aging of HSCs and the whole hematopoietic system in elderly, or even achieve a certain degree of their rejuvenation? Several important experimental studies conducted in animals indicate that this seems to be a real possibility.

In concordance with the notion that aging HSCs are characterized by enhanced metabolism and disturbed mitochondrial function, a recent study demonstrated that continuous supplementation of aged mice with nicotinamide riboside restores mitochondrial function and metabolic capacity in HSCs, establishing a more youthful hematopoietic system [193].

Recent introduction of senolytics, agents that are able to selectively eliminate senescent cells in the aging organism, raised significant hopes that they might be instrumental in delaying organismal aging. Indeed, a recent work demonstrated that rejuvenation of the hematopoietic system in aging mice might be achieved with senolytic drug ABT263 [194].

Since elevated activity of small RhoGTPase CDC42 has been shown to produce loss of polarity and aging phenotype in HSCs [182], one of the potential approaches to rejuvenation of the latter is to inhibit CDC42 activity. In concordance with that, pharmacological inhibition of CDC42 ex vivo rejuvenates aged HSCs [182]. However, the activity of these ex vivo rejuvenated HSCs in the old mice is restrained by the aged BM niche [195]. On the other hand, as short-term treatment of aged female mice with CDC42 inhibitor significantly extends their lifespan [196], one may hypothesize that inhibition of this GTPase partially restores function of the niche as well.

Yet another aging mechanism is related to protein quality control. In a very recent study, chaperone-mediated autophagy (CMA), one of the three main forms of autophagy, was shown to play essential role in HSC function in mice. CMA activity declines in HSCs with age, whereas its genetic or pharmacological activation restores the functionality of aging HSCs [197], demonstrating the possibility of rejuvenation of HSCs by targeting CMA.

A recent study revealed that degeneration of the sympathetic nervous system innervation in the BM niche is an important factor driving aging of HSCs. Accordingly, supplementation of old mice with adrenoreceptor β3-selective sympathomimetic resulted in significant restoration of the in vivo function of aging HSCs [198].

One of the conceptually promising strategies to achieve hematopoietic rejuvenation is a heterochronous transplantation of young HSC or MSCs to elderly. Some earlier results indicated that infusion of young bone marrow cells or MSCs significantly extended the lifespan of mice [199,200]. Based on these and other findings, Rožman proposed extending healthy human life span by autologous transplantation in aging individuals of cryopreserved HSCs collected during their young age [201]. Apart of the questionable feasibility of this strategy in the coming years, the latest works in this field raised certain doubts as to whether this eventually proves to be an efficient approach as far as the hematopoietic system itself is concerned. In particular, aged HSCs transplanted to young animals were unable to restore their functionality in the young BM environment [202,203]. On the other hand, young HSCs were largely unaffected by aging environment [203], which raises hopes that they might rejuvenate the aging hematopoietic system. In this respect, a cautionary note was sent by the study in which the function of transplanted ex vivo rejuvenated HSCs was limited by the aging niche [195]. Whether the same applies to the naturally young HSCs remains to be seen.

Thus, the heterochronous transplantation of young BM stem cells apparently needs more work to elucidate whether it might be efficient for rejuvenation of the hematopoietic system. However, several other studies demonstrate convincingly that this approach is able to alleviate functional decline of other aging tissues and to achieve overall extension of lifespan in mice [204,205].

## 6. Translation to the Clinic

As most of the research data described in this review were obtained in the few last years, they are only starting to move toward the clinic. We summarize below a few examples of how the recently established knowledge about the hematopoietic system is being advanced to the clinic.

High occurrence of asymptomatic clonal hematopoiesis (CH) in elderly, its high impact on the incidence of cardiovascular diseases, and the serious risks associated with its progression to leukemia [191] represent a serious challenge to healthcare systems that needs to be addressed. Apparently, the early diagnostics of CH would allow risk stratification and focused monitoring of afflicted individuals for timely detection of adverse developments. The fact that only a limited number of genes, among which DMT3A, TET2, and ASXL1 are the most frequent, are mutated in CH, makes the goal to develop robust and inexpensive initial diagnostics of CH fairly feasible.

Notably, TET2, which is frequently associated with CH, negatively regulates HSC function and myelopoiesis, and its activity depends on vitamin C (ascorbate). Ascorbate supplementation in vivo restores defective function of TET2 [206,207]. This finding provides a potential therapeutic avenue for patients with CH transitioning to leukemia, and a general strategy to suppress hyperactive HSCs. In support of this, combination of ascorbate with decitabine had positive effect on remission rates and survival in patients with acute myeloid leukemia compared to decitabine alone [208]. Common for CH is an elevated level of pro-inflammatory cytokines, in particular interleukin 1 beta (IL-1β), which increases risk of cardiovascular complications. In CANTOS trial, therapy with IL-1β-neutralizing antibody canakinumab significantly decreased the risk of cardiovascular events [209]. Since SIRT1 deacetylase enhances activity of TET2, another potential way to limit adverse effects of CH is to use SIRT1 activators such as SRT1720 or resveratrol [210,211].

The 2005 discovery that Philadelphia chromosome-negative myeloproliferative neoplasms, and most prominently polycythemia vera (PV), are associated with activating V671F mutation in JAK2 kinase [212,213], opened the way for development of new strategies based on JAK2-specific antagonists for treatment of these conditions. Experimental studies revealed that supplementation with JAK2 inhibitors ruxolitinib or fedratinib reduced HSPC proliferation and myelopoiesis, inhibited production of several pro-inflammatory cytokines, and reduced the risk of atherosclerosis in animals [214,215]. Subsequent clinical RESPONSE trial with ruxolitinib treatment of PV demonstrated its efficacy in disease modification and reduction of symptoms burden [216]. This agent has been approved by FDA and is used as a second-line drug for PV therapy, especially for hydroxyurea-resistant or intolerant patients. Among other agents showing promising results in PV trials are the histone deacetylase inhibitor givinostat and PTG-300 and subcutaneous hepcidin mimetic (reviewed in [217]).

One of the important goals of the focused studies of the last years was the identification of substances and conditions able to expand HSCs ex vivo for clinical use, in particular transplantation. It seems that extensive research in this field has brought finally a sizeable success with umbilical cord blood (UCB) transplants. UCB transplants represent an attractive alternative to BM, but are often plagued by delayed and suboptimal engraftment resulting in transplant-related mortality. The use of StemRegenin–1, aryl hydrocarbon receptor antagonist, demonstrated in a small-scale trial that cotransplantation of expanded and nonexpanded cord blood units significantly reduced time to neutrophil recovery, and that expanded unit was engrafted in all patients [218]. A fairly similar approach was used for testing efficiency of nicotinamide (NAD). As in the above example, significant reduction in neutrophil recovery time and successful long-term engraftment of expanded units in majority of recipients was achieved [219]. A phase I–II trial demonstrated that transplantation of NAD-expanded UCB units only results in full and broad immune response comparable the one achieved with conventional graft sources [220]. Recently published results of phase III trial using transplantation of standalone NAD-expanded units confirm earlier conclusions on significant reduction of time for neutrophil and platelet recovery compared to control, and reduced incidence of infections. No statistically significant differences with control were observed for recipient survival and graft-versus-host disease (GvHD) [221]. Current studies on ex vivo expansion of HSCs and the available results of clinical trials are described in more detail in a recent review by Zimran et al. [222]. It should also be noted that the successful expansion of long-term engrafting HSCs from UCB paves the way to development of advanced strategies of HSCs-based gene therapy using DNA-integrating vectors or genome editing.

Since MSCs have potent immunomodulatory properties, a number of clinical trials tested whether they can be instrumental in alleviating GvHD, a serious, potentially lethal complication during HSCT (HSC transplantation). Two meta-analyses of existing results revealed that MSCs infusion generally improved engraftment and reduced chronic GvHD incidence, especially for cord blood-derived MSCs, whereas infusion of BM MSCs had no positive effect. MSC cotransplantation was more effective in children and young individuals with HLA-nonidentical HSCT [223,224]. These studies thus support the application of MSCs as a strategy to reduce the risk and severity of GvHD during HSCT.

Significant numbers of other clinical trials are dealing with the use of MSCs’ immunomodulatory properties as standalone therapy for various diseases and conditions associated with overactivated immune systems. MSCs were tested for treatment of rheumatoid arthritis and osteoarthritis [225], multiple sclerosis [226], inflammatory bowel disease [227], systemic lupus erythematosus [228], diabetes mellitus [229], among other pathologies. In most reported cases, good safety profiles and encouraging outcomes were reported. However, these studies are still in their early stages and more clinical trials showing safety and efficacy are apparently required.

Substantial interest represents the use of stem cell transplantation for treatment of neurological and neurodegenerative diseases. Numerous experimental studies demonstrated that cord blood infusions stimulate neurogenesis in the aged brain and are therapeutically efficient in the treatment of ischemic stroke, traumatic brain injury, amyotrophic lateral sclerosis, Alzheimer’s disease, and Parkinson’s disease (reviewed in [230]). A recent phase I clinical trial, which demonstrated that infusion of non-HLA-matched allogeneic UCB into ischemic stroke patients was safe and resulted in health improvements [231], opens the door for phase II and III clinical trials using UCB for stroke therapy and, potentially, other diseases of the nervous system.

## 7. Concluding Remarks

The development, functioning, and age-related modifications in the hematopoietic system are extremely complex processes that require the involvement of many different cells, an enormous number of substances they produce, and, most importantly, a myriad of molecular and functional interactions between the participants. This seems to function like a grandiose orchestra, where each instrument leads its own party while all together merge into a polyphonic musical composition of exquisite beauty. During the past 100 years, we have learned much about the structure and functioning of the hematopoietic system. However, we do not yet know many important details necessary for understanding the orchestration of the entire process. In particular, knowledge of numerous molecular players together with essential epigenetic regulation of hematopoiesis is fragmentary at best, and it still requires much effort to fill even the largest gaps. Nonetheless, one may hope that in the coming years, using the latest advances in molecular cell biology and equipped with extremely powerful analytical tools developed recently, we will be able to decipher a nearly complete “musical score” of the true nature’s masterpiece named hematopoiesis. This in turn will greatly promote our unending quest for new molecules and medical technologies that could advance the treatment of numerous pathologies and improve the quality of human life.

## Figures and Tables

**Table 1:** Changes in activated HSCs in comparison with quiescent ones, adapted from [63].

Increased in Comparison with Quiescent Stem Cells	Decrease in Comparison with Quiescent Stem Cells
Cell size	Bivalent domains *
Histone methylation	Translation repression
RNA content	Autophagy
Protein synthesis	
Metabolism	

* chromatin domains associated with both repressing and activating epigenetic regulators.

**Table 2:** Relationship between intrinsic and extrinsic mechanisms associated with aging of hematopoiesis and immune function [105].

	Increased in Comparison with Young	Decreased in Comparison with Young	Altered Factors
Intrinsic factors	Predominance of myeloid-biased HSC and gene expression changes Oligoclonality Somatic mutations DNA damage markers	Cell cycle	Epigenetic drift
Extrinsic factors	Mature myeloid cells Chronic inflammation	Immune function	Local cytokines and growth factors Cellular composition

**Table 3:** Aging-related changes in HSC, adapted from [121].

Increased in Comparison with Young	Decreased in Comparison with Young
Distance to the endosteum	Proliferative potential
Predominance of myeloid differentiation	BM homing
Mobilization by cytokines	

**Table 4:** Aging-related changes in the mouse BM niche, adapted from [74].

Cells	Increased in Comparison with Young	Decreased in Comparison with Young
Endothelial cells	Vascular density Vascular permeability Intracellular ROS.	The number and length of arterioles and type H capillaries Expression of CXCL12, Jagged 1, and SCF.
BM MSC	Adipogenesis Number of BM MSCs	Osteogenesis The number of osteoprogenitors The number of perivascular cells αSMA+ PDGFRβ + cells and NG2 + cells CFU-F capacity and expression of niche HSC factors
	Changes in the composition of secreted exosomes Epigenetic changes	
Compact Bone MSCs		The number of CFU-Fs associated with bone cells Nes-GFP +
Osteogenic cells	Expression of CCL5	OPN expression The number of CD45-TER119-CD31-SCA1-CD51 + cells enriched in osteoblasts
Adipocytes	The number of adipocytes	
Nerves		The number of adrenergic and tubulin-βIII-positive nerve fibers The number of synaptophysin-positive synapses
Megakaryocytes	The number of megakaryocytes and megakaryocyte precursors	
